# The effects of precipitation change on urban meadows in different design models and substrates

**DOI:** 10.1038/s41598-023-44974-y

**Published:** 2023-11-23

**Authors:** Yarong Jiang, Tao Yuan

**Affiliations:** 1 College of Horticulture and Forestry Science, Huazhong Agricultural University, Wuhan, 430070 China; 2 School of Landscape Architecture, Beijing Forestry University, Beijing, 100083 China; 3 National Engineering Research Center for Floriculture, Beijing, 100083 China; 4 Beijing Laboratory of Urban and Rural Ecological Environment, Beijing, 100083 China

**Keywords:** Ecology, Climate sciences, Ecology, Environmental sciences

## Abstract

Climate change, such as temperature and precipitation changes, is becoming increasingly obvious, and in this context, planting designs need to urgently consider future climate change in advance. A field experiment was conducted in Beijing, China, where the future precipitation is predicted to increase, and extra irrigation was used to simulate the future precipitation increase. The species richness of sown meadows, including spontaneous plants and sown plants, and the adaptive strategies of the communities were recorded under different types of design models and substrates. The results showed that precipitation increased the diversity of sown plants and resource-demanding spontaneous plants but had no significant effect on the dry matter content of the entire community of species. Moreover, the interactions among precipitation and substrate, especially the design models, were significant. Of the models, the three-layer model had the highest species richness and least invasive plants. In addition, increased precipitation significantly changed the functional strategy of the plant community away from ruderals and towards competitor-stress tolerant species. This study provides guidance for the design and management of naturalistic plant communities under climate change.

## Introduction

Climate change is becoming increasingly obvious, and it is well known that the temperature has increased over the past few decades^1^. Unlike the observed and predicted global temperature increases, precipitation projections are more uneven in the future^2^, for example, there is a general wetting trend in arid regions and a drying trend across humid regions in China^3^. Moreover, it is predicted that two-thirds of the Earth’s land area will become wetter^4^, and precipitation frequency and amount will increase in these areas, which means a risk of flood disasters^5,6^.

Changes in precipitation caused by climate change have influenced plants in many ways, including phenological changes^7,8^, driving substantial changes in plant coexistence^9^, decreased biomass^10^ and increased pest infestation^11^, even threatening their growth and causing death^12^. In urban areas, the effects of precipitation change might be enhanced because soil sealing and building materials intensify water loss and stress, and plants may not be able to cope with future precipitation change^13,14^. Moreover, changes in precipitation deeply affect the irrigation frequency for plants. Therefore, it is essential to consider the challenges of future precipitation change in planting designs.

Naturalistic plant communities are considered more resilient and sustainable under harsh conditions^15–18^. These communities inspired from natural grassland, are not only present a beautiful landscape for the public but also serve as habitats for spontaneously occurring plants and animals^19,20^, which is beneficial for increasing biodiversity. By simulating precipitation, temperature and CO_2_ concentration changes, Alizadeh and Hitchmough^21,22^ found that a mix of maritime climate species and southern European and Mediterranean climate species showed a high level of sustainability in current and future UK climate scenarios. Nagase and Dunnett^23^ showed that under sufficient water conditions, plant communities at low densities grow well; when high-density vegetation is not watered, community species diversity is rich. These results imply that there is an interaction between water supply and plant community features. Hitchmough et al.^24^ proposed a two-layer perennial ‘prairie-meadow’ vegetation design that resulted in flowers that were diverse and resistant to weed colonization and, more importantly, did not need extra irrigation. Thus, different design models of plant communities may respond differently to precipitation changes.

Design models that involve different plant heights and canopy layers have different appearances and plant and soil microbial diversity^20^. Hitchmough^25^ summarized three design models with different combinations of species (Table 1). Based on observations of natural pictorial meadows in northern China, Fang et al.^26^ revealed another three-layer plant community design model; however, it is unclear what the adaptation potential and fitness of these different design models are in response to climate change.

**Table 1:** Design models of the naturalistic plant community.

Design models	Canopy layers	Characteristics	References
Single layer	Single layer	Composed of species with similar heights and growth potentials	Hitchmough^25^, Hitchmough et al.^24^
Shade-tolerant lower layer and taller emergent layer	Two layers	Base layer covers the ground, can persist in heavy shade. Most species in the upper layer have leafless flowering stems, and the emergent density generally does not exceed 10 percent of the total seeding density	
Two-layer systems with an extra layer	Three layers	Extra layer contains a few large forbs. Species richness is extra layer˂emergent layer˂lower layer	
Taller layer + clustered second layer + creeping lower layer	Three layers	Erect with no or few branches in tall layer, cespitose plants in second layer, stoloniferous or rosette in lower layers. The species richness is taller layer˂lower layer˂second layer	Fang et al.^26^, Fang et al.^27^

Moreover, different substrates vary in their water retention properties, which may influence plant growth^28^ and the development of meadows^29^, while soil type has an even greater effect than management treatment^30^. Sand-mulched substrates minimize weed germination and competition, and topsoil is the most weedy layer^30,31^. However, the joint interactions of all three (precipitation change, design models, and soil substrates) remain underexplored.

As the capital city of China, Beijing is one of the largest and most rapidly-urbanizing cities in the world, and the vegetation faced with the challenges of biodiversity loss and climate change, accordingly, it is vigorously advocated by the government to protect urban biodiversity, such as increasing the species diversity of spontaneous plants in cities, and implement adaptive planting measures to cope with climate change^32^. In addition, the Inner Mongolia region where was adjacent to Beijing has a large natural distribution of grassland, and a large number of famous poets and artists in ancient China have praised the beauty of the grassland landscape. Moreover, there was once a large natural grassland in the Shimadai imperial garden of Chengde Mountain Resort near Beijing for aristocrats to enjoy and hunt^33^. Therefore, naturalistic plant communities is preferred and promoted in Beijing.

We designed and established naturalistic plant communities in Beijing, which has a typical temperate continental monsoon climate, with an average annual temperature of 11.7 °C. Beijing is hot and rainy in the summer, with more than 70% of its annual precipitation occurring in the summer, but the winter is cold and dry^34^. Moreover, the average annual precipitation in Beijing is approximately 600 mm^35^. According to the projections, the frequency of precipitation in Beijing will increase, and the annual total amount of precipitation will increase at an approximate rate of 9 mm/10 years under the Representative Concentration Pathway (RCP) 4.5 emission scenarios^3,36,37^. In brief, climate change will lead to a wetter Beijing. Moreover, the designed plant communities in the city are not preferred by the public, as they prefer natural meadows^33^.

Thus, natural spontaneous plants have been highlighted, as plant species that are not sown and are classified as invasive and resource-demanding plants according to Oudolf and Gerritsen^38^. Invasive plants usually dominate large areas, and these species must be restrained and prevented and are usually considered ‘weeds’. While resource-demanding plants are familiar and well-loved specimens, they also can contribute to the natural beauty of communities and do not have to be removed.

By increasing the irrigation amount to simulate the increase in future precipitation and by establishing naturalistic plant communities using different design models and substrates, we attempted to assess species richness and the state of plant communities based on their life strategies (Grime’s Competitiveness (C), stress tolerance (S), and ruderality (R) (CSR theory)), as this approach has been used to visualize the trajectory of succession in communities and understand vegetation dynamics^39,40^, to study (i) the effects of increasing precipitation on the richness and adaptive strategies of herbaceous communities (including sown plants and spontaneous plants) and determine (ii) whether the design models and substrates are conducive to mitigating the risk of precipitation change?

## Methods

### Study area

The field experiment was conducted on the nursery grounds of the M-Grass Beijing Water Efficient Landscape Architecture Technology Company Ltd, Beijing, China (39°44ʹ9ʹʹN, 116°44ʹ39ʹʹE). The experimental area had previously been planted with *Lilium brownii* var. *viridulum*, and the soil was sandy loam with few weeds. In the autumn of 2017, a rotary tiller was used to plough and remove the lily bulbs, and the field was ploughed again in March 2018.

**Figure 1:**
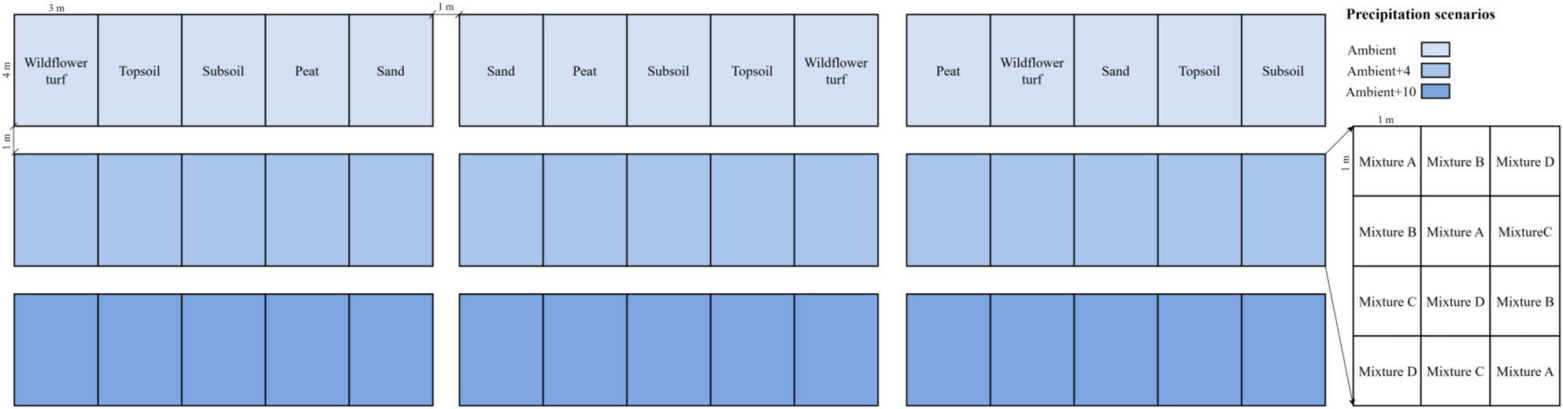
Layout of the experimental plots.

A split-plot design (Table 2, Fig. 1) was adopted, and precipitation was set as the first-level experimental factor (main treatment). The substrates and design models were two-level and three-level experimental factors, respectively (secondary treatment). At least a 1 m wide isolation buffer zone was set in the main treatment, and the plots were separated by 30 cm wide field ridges. Each block was assigned to different soil substrate treatments, and the topsoil treatment was set as the control. Then, plots (1 m × 1 m) in the block were randomly assigned to the different design models (Fig. S1). The design of the sown plant communities is shown in Table 3, and every treatment had three replicates in the experiment.

**Table 2:** Split-plot design in the experiment.

Factors	Level	Details
Precipitation scenarios	Ambient	No additional watering except for sleet in winter and regreening water in early spring
	Ambient + 4	Watered 4 × 9 mm to simulate the amount of precipitation after 40 years, in addition to the sleet and regreening water
	Ambient + 10	Watered 10 × 9 mm to simulate the amount of precipitation after 100 years, in addition to the sleet and regreening water
Design models	Mixture A	Single layer, composed of species with similar heights and growth potentials
	Mixture B	Two layers, and the density in the emergent layers did not exceed 10 percent of the total seeding density
	Mixture C	Three layers, most species and an extra layer with a few tall forbs, and the species richness was extra layer˂emergent layer˂lower layer
	Mixture D	Three layers, the species richness was taller layer˂lower layer˂second layer
Substrates	Wildflower turf	Peat and vermiculite was mixed at a volume ratio of 3:1 and cultivated in greenhouse
	Topsoil	Depths of the ploughed soils were approximately 10 cm
	Subsoil	Removed a 10 cm depth of surface soil and ploughed the soil to approximately 10 cm
	Peat	Covered the field with a 5 cm depth of river sand and then a 5 cm depth of peat in the upper layer. The peat used was sterile, arable, moist, water-retaining, with high fertility, the brand of peat was ‘Flora gard’ which was imported from Germany
	Sand	Covered the field with a 10 cm depth of river sand which had good permeability and was easy to drain

**Table 3:** Sowing rates used in the experiment.

No.	Species	1000-seeds weight (g)	Germination rate (%)	Canopy height	Sowing rate (g/m^2^)			
					Mixture A	Mixture B	Mixture C	Mixture D
1	*Chamaemelum nobile*	0.1	96.7	Low	0.03			
2	*Potentilla chinensis*	0.13	77.3	Low	0.05	0.05	0.05	0.05
3	*Linaria vulgaris*	0.07	73.3	Low	0.05	0.05	0.05	
4	*Allium giganteum*	0.12	92.2	Low	0.1	0.1	0.01	0.2
5	*Nepeta cataria*	0.8	73.3	Low	0.1	0.1	0.1	0.05
6	*Achillea sibirca*	0.52	72.0	Low	0.1	0.1	0.1	0.1
7	*Dianthus chinensis*	0.84	73.3	Low	0.1	0.1	0.1	0.2
8	*Oenothera speciosa*	0.16	48.3	Low	0.1	0.1		
9	*Papaver nudicaule*	0.14	33.3	Low	0.1	0.15	0.15	0.15
10	*Trifolium repens*	0.06	85.3	Low	0.1			0.1
11	*Chrysanthemum multicaule*	1.5	30.7	Low	0.15	0.3	0.15	
12	*Saponaria officinals*	0.91	45.6	Low	0.2	0.2	0.2	
13	*Linum usitatissimum*	1.5	85.3	Low	0.2			
14	*Scabiosa comosa*	6.1	61.1	Low	0.25	0.25	0.25	0.5
15	*Plantago asiatica*	0.57	54.4	Low	0.3	0.3	0.3	0.3
16	*Viola philippica*	1.1	28.0	Low	0.3			0.3
17	*Hyssopus officinalis*	0.97	42.2	Medium	0.1	0.1	0.1	0.1
18	*Salvia nemorosa*	1	75.6	Medium	0.1	0.1	0.1	0.2
19	*Veronica didyma*	0.07	52.0	Medium	0.1	0.1	0.1	
20	*Monarda didyma*	0.39	46.7	Medium	0.1	0.1		
21	*Callistephus chinensis*	0.18	52.2	Medium	0.2	0.1	0.2	0.2
22	*Lychnis fulgens*	0.44	45.3	Medium	0.2			0.3
23	*Allium senescens*	1.5	37.3	Medium	0.3			
24	*Echinacea purpurea*	3.8	65.6	Medium	0.8	0.5	0.5	
25	*Cerinthe major*	50.6	82.2	Medium	1	2		1
26	*Oxytropis bella*	3.8	17.8	Medium	1.3			1.3
27	*Physostegia virginiana*	2.5	16.0	Medium	2			
28	*Cheiranthus allionii*	1.3	8.9	Medium	2.9	4.4	4.4	4.4
29	*Lolium perenne*	1.7	20.0	Medium		0.2	0.2	0.5
30	*Rudbeckia hirta*	0.6	88.0	Tall		0.05	0.05	0.1
31	*Leucanthemum vulgate*	3.9	44.4	Tall		0.1	0.1	0.2
32	*Eryngium agavifolium*	1.4	76.7	Tall		0.2	0.2	0.6
33	*Gomphrena globosa*	3.9	51.1	Tall		0.4	0.5	0.8
34	*Coreopsis basalis*	1.4	17.3	Tall		0.4		
35	*Anethum graveolens*	2.1	14.4	Tall		2	2	4
36	*Consolida ajacis*	2.8	18.7	Tall		3	3	3
37	*Glandularia canadensis*	0.99	0.00	Tall			0.3	0.5
38	*Geum aleppicum*	1.4	17.3	Tall			0.4	0.4
39	*Verbena bonariensis*	0.24	9.3	Tall			0.4	0.6
40	*Lupinus perennis*	23	84.0	Tall			0.5	0.5

The germination rate was tested at a constant temperature of 20 °C in January and February 2018, and the light and dark treatments were both 12 h. Seeds were purchased from the Beijing Forestry University Science Company and Lvzhiyuan Seed Industry Company in Gansu Province, China.

The seed mixtures were sown evenly when there was no wind. At the same time, with reference to the method for wildflower turf in Hewetson-Brown^41^, two layers of a coconut fibre blanket were placed on the 45 cm × 45 cm × 7 cm seedling plate in the Beijing Forestry University Science Company’s Greenhouse (40°0ʹ38ʹʹN, 116°20ʹ41ʹʹE) and then covered with 5 cm thick substrates (peat and vermiculite). Finally, the seeds were mixed with wet sand and evenly spread in March 2018 for several days to grow and were transferred to the experimental field on April 25 2018 (Fig. S1).

### Precipitation scenarios

**Figure 2:**
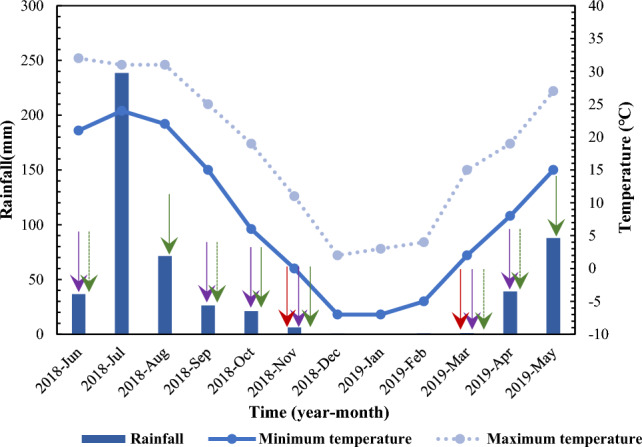
Mean monthly temperature and rainfall changes during the experimental period in Beijing. The rainfall data were summarized from the Dongfangxiangyun Big Data Platform (http://www.dfsjcloud.com), and the temperature data were obtained from the Weather Website (http://www.tianqi.com). The red, purple and green arrows indicate the watering period corresponding to the Ambient, Ambient + 4, and Ambient + 10 treatments, respectively. When the Ambient + 10 treatment was watered twice in a month, the green arrow was a dotted line.

From June to the end of October in 2018 and early March to May 29th, 2019, we avoided rainy days (Fig. 2); used a measuring cup (maximum volume was 9 L), bucket and watering bottle to irrigate; and ensured that approximately 9 L/m^2^ of water was in the treatments. The substrate could be fully irrigated but seldom drained off, which was equivalent to 9 mm precipitation every application. The Ambient + 4 treatment received a total of 36 mm of precipitation in addition to sleet and regreening water (Fig. 2), which was necessary in Beijing, and the amount of precipitation simulated the rainfall change after 40 years. The Ambient + 6 treatment simulated the precipitation increase after 100 years.

### Data collection

At the end of May 2018, a quadrat (20 cm × 20 cm) survey was conducted to record the species and seedling abundance (including sown species and spontaneous species) in all treatments, and the survival rate was determined. And before the quadrat survey in May 2018, we tested 6 sample sizes including 10 cm × 10 cm, 20 cm × 20 cm, 30 cm × 30 cm, 40 cm × 40 cm, 50 cm × 50 cm, 100 cm × 100 cm to calculate the species-area curves, and found that 20 cm × 20 cm could cover most of species in the plot and even though the sample size increased over 20 cm × 20 cm, the species diversity was stable. In addition, the plant density was very high in the seedling stage and to ensure the accurate count of plant abundance, 20 cm × 20 cm quadrat was determined. Because the meadows were sown in spring when weeds easily invade and need to be controlled, invasive and capricious plants were classified as invasive weeds. Then, the invasive plants were recorded and removed every other month, packed in envelopes and dried in an oven at 80 °C to constant weight, and the dry matter content was recorded.

**Figure 3:**
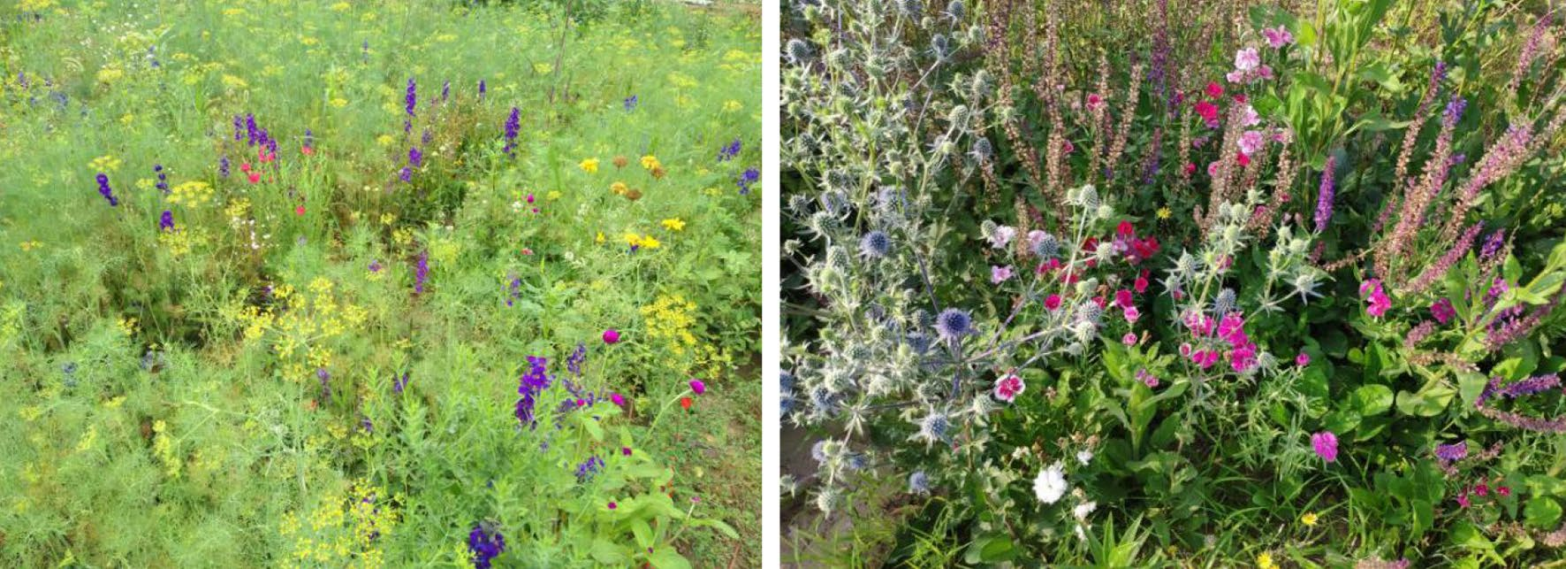
Plant community in 2018 and 2019.

In September 2018, the vegetation was surveyed again with a quadrat size of 30 cm × 30 cm based on the species-area curve method as above. The species and abundance of sown plants and resource-demanding plants in the community were recorded. And the aboveground parts were harvested in November 2018. Then, in June 2019, quadrat samples (30 cm × 30 cm) were used to investigate the species and abundance of sown species and resource-demanding plants again, with 4–10 samples in each treatment to ensure that all species were identified. All aboveground parts were harvested after the vegetation survey, and the dry matter content of all species in each treatment was measured. In particular, the dry matter content of the invasive plants was the cumulative value from June 2018 to June 2019. And the research and field studies on plants (either cultivated or wild), including the collection of plant material, complied with Beijing, China, and international guidelines and legislation. In addition, flower meadow growth was observed every other week, and pictures were taken to record the appearance of the vegetation. The maximum and minimum temperatures and precipitation in the Beijing area for each month in 2018 and 2019 are shown in Fig. 3.

### Data analysis

The species richness of the community was the number of species in a quadrat. A general linear model was used for multifactor analysis of variance in SPSS version 26, and Duncan’s test was used for multiple comparisons for significant differences. The main effects and interactions of the three experimental variables in the community in 2019 were analysed. The three variables were fixed factors, and the blocks were random factors. Multiple comparisons were made in SPSS. In addition, all data generated or analysed during this study are included in this published article and its supplementary information files.

To determine the effect of precipitation change on the adaptive strategies of plant communities, species were labelled with functional traits referring to the CSR strategy plant list^42,43^ and the method of Thuring^44^ and then drawn from the online database Plant Science Data Center (http://www.iplant.cn/). The relative abundance was calculated based on plant data obtained by the vegetation survey, and the CSR signature, standard coordinate values in the space, number of CSR types, and CSR distance between different communities were all calculated in the CSR Signature Calculator from UCPE Sheffield^42,45^. Finally, based on the coordinates of the community, the ggtern package was downloaded in R 4.2.1 to draw ternary diagrams to reveal the changes in the adaptive strategies of the different communities. Moreover, packages including ggplot2, ggpubr, ggsignif, tidyverse, and ggprism were downloaded in R for multiple comparisons of CSR scores among the different irrigation treatments, and box plots were drawn.

## Results

### Plants in the community

In May 2018 (Fig. 3, Fig. S1), approximately 93% the sown plant species were observed in the field, and the dominant species were *Anethum graveolens*, *Linaria vulgaris* and *Consolida ajacis*, the richness values of the plant communities were from 13 to 21, which have no significant differences among treatments (*P* > 0.05). Spontaneous plants, including 7 resource-demanding species and 9 invasive plants were found in 2018 (Table S1). In the second year (Fig. 3), *Salvia nemorosa* and *Eryngium agavifolium* were dominated in the community, and there were 6 resource-demanding species and 8 invasive species, although the field was intensively managed, *Aster novi-belgii* was observed very often and came from surrounding field. Some resource-demanding plants, such as *Taraxacum mongolicum*, *Inula japonica* and *Ixeridium sonchifolium*, are native plants with wild beauty, and are usually located in the lower and second layers. In contrast, 54.5% of the spontaneous plants were invasive species, and *Setaria viridis* was the most common weed which was located in the second and taller layers of the community, occupies the main dry matter weight in the second year. Invasive perennials, *Cirsium arvense* var. *integrifolium* and *Cynanchum chinense,* for example, are common farmland weeds in Beijing. Shrubs such as *Salix caprea* were also found in the field, and they were difficult to remove and competitive species. Moreover, the landscape of naturalistic plant communities under different precipitation scenarios was similar. And substrates and design models had great influence on community appearance. The landscape of wildflower turf was significantly different from other treatments, and mixture A was lower than other seed mixtures.

### Species richness changes

Precipitation scenarios had a significant effect on community diversity (Table 4, *P* = 0.00), with the increase in precipitation promoting the richness of sown species and resource-demanding plants in the second year, and the average species richness of sown species and demanding plants per quadrat in ambient precipitation scenarios was 3.3 (the standard deviations was 1.7), while ambient + 10 precipitation scenarios was 4.2 (Table 5). Design models and substrates also had significant effects on the richness of sown species and resource-demanding plants that needed to be protected (*P* = 0.00); plants in the topsoil and subsoil had the highest diversity (4.2 and 4.6) compared to those in other substrates. There were obvious interactions among the three variables (*P* = 0.01), but there was no interaction between precipitation and substrates (*P* = 0.98), which means it is feasible to adapt the influence of precipitation change on species richness through plant design and substrates, yet substrates can interact with precipitation only under certain design models. Communities with more water and multilayer communities (Mixture C and Mixture D) were the most diverse, and the species richness was 4.5 and 4.0, while the single-layer mixtures had the lowest diversity.

**Table 4:** Significance test of precipitation scenarios, design models and substrate influences on species richness.

Variables	Species richness of sown species and resource-demanding plants	
	F value	*P* value
Precipitation scenarios	16.7	**0.00**
Design models	16.8	**0.00**
Substrates	27.5	**0.00**
Precipitation scenarios × Design models	2.8	**0.01**
Precipitation scenarios × Substrates	0.15	0.98
Precipitation scenarios × Design models × Substrates	2.2	**0.01**

Numbers in bold indicate that the influences were significant (*P* < 0.05); the same is true below.

**Table 5:** Multiple comparisons of precipitation scenarios, design models and substrate influences on species richness.

Variables	Level	Species richness of sown species and demanding plants
Precipitation scenarios	Ambient	**3.3a**
	Ambient + 4	**4.0b**
	Ambient + 10	**4.2b**
Design models	Mixture A	**2.8a**
	Mixture B	**3.7b**
	Mixture C	**4.5c**
	Mixture D	**4.0b**
Substrates	Wildflower turf	**2.4a**
	Topsoil	**4.4d**
	Subsoil	**4.6d**
	Peat	**3.7c**
	Sand	**3.1b**

The values in the table are the average values of each treatment. The different lowercase letters after the average value in the same group indicate that the data showed significant differences after multiple comparisons. It is same in the Table 7.

Significant values are in [bold/italics].

### Dry matter content change

Precipitation had little significant impact on the dry matter content of the three types of plants in the community (Table 6, *P* > 0.05). With the increase in precipitation, the dry matter content of the invasive plants increased indistinctively, while the dry matter content of sown plants decreased slightly (Table 7). And substrates and design models had significant effects on invasive plants, but precipitation scenarios had a significant interaction with substrates and especially in the design models (Table 6), which indicated that precipitation scenarios had indirect effects on dry matter accumulation of plant communities on different design models and substrates. Mixture D with the wildflower turf and no additional watering had the highest dry matter content, and the combination of Mixture A, sand and high precipitation resulted in a lower dry matter content of the sown plants (0.57 ± 0.58) and invasive weeds (17.2 ± 3.0).

**Table 6:** Influences of precipitation scenarios, design models and substrates on the dry matter content.

Source of variation	Invasive plants		Resource-demanding plants		Sown plants	
	F value	*P* value	F value	*P* value	F value	*P* value
Precipitation scenarios	2.3	0.21	2.0	0.24	0.16	0.86
Design models	18.6	**0.00**	1.8	0.15	20.1	**0.00**
Substrates	7.6	**0.01**	2.6	0.19	3.2	0.08
Precipitation scenarios × Design models	8.8	**0.00**	1.0	0.4	0.77	0.60
Precipitation scenarios × Substrates	3.8	**0.03**	2.3	0.12	1.3	0.34
Precipitation scenarios × Design models × Substrates	6.2	**0.00**	1.3	0.22	1.0	0.45

Significant values are in [bold].

**Table 7:** Multiple comparisons of the dry matter content (g) of the communities under different precipitation scenarios, design models and substrates.

Variables	Level	Invasive plants	Resource-demanding plants	Sown plants
Precipitation scenarios	Ambient	6.8	0.26	44.3
	Ambient + 4	7.6	1.89	42.0
	Ambient + 10	11.6	1.1	33.6
Substrates	Wildflower turf	**1.2a**	4.9	73.9
	Topsoil	**3.4a**	0.02	51.5
	Subsoil	**5.2ab**	0.54	49.9
	Peat	**13.6c**	0.76	29.9
	Sand	**10.7bc**	0.24	25.2
Design models	Mixture A	**15.6a**	1.5	**16a**
	Mixture B	**8.1b**	2.0	**33.2b**
	Mixture C	**4.4b**	0.13	**50.5c**
	Mixture D	**5.1b**	0.00	**64.1c**

Significant values are in [bold].

### Adaptive strategy changes

The invasive plants were competitive and weedy species, and most of them were tall. While approximately 50% of the resource-demanding species were stress tolerators, usually located in the lower and second layers. In the fall of 2018, the dominant species of Mixture A and Mixture B became *Oenothera speciosa* (R/CSR), the vegetation diversity was poor, and there was bare ground. In mixture D, *Salvia nemorosa* (CR) was the dominant species. By the second year, almost all communities were dominated by *Eryngium agavifolium* (C) and *Salvia nemorosa*, and some species, including those dominant in different precipitation treatments, were different and tended to compete; both Mixtures A and D on peat were occupied by *Trifolium repen* (CR/CSR), covering the ground with creeping stems and green leaves. Only a small number of sown plants survived, and vegetation diversity was very low.

**Figure 4:**
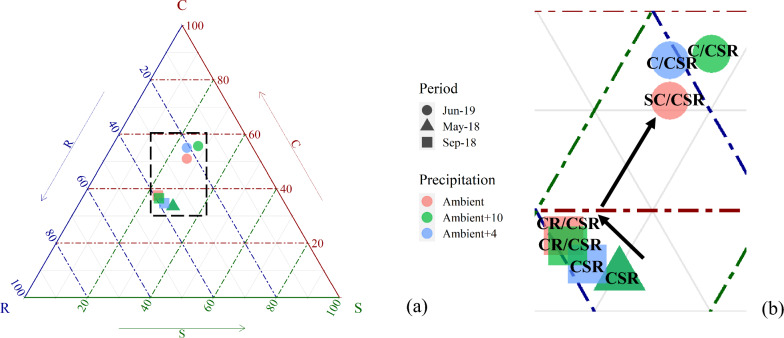
Functional character of the vegetation under different precipitation scenarios. (**b**) Enlarged images from the area circled by a black dotted line in (**a**), and the black arrows show the trends in the community strategies in different periods.

**Figure 5:**
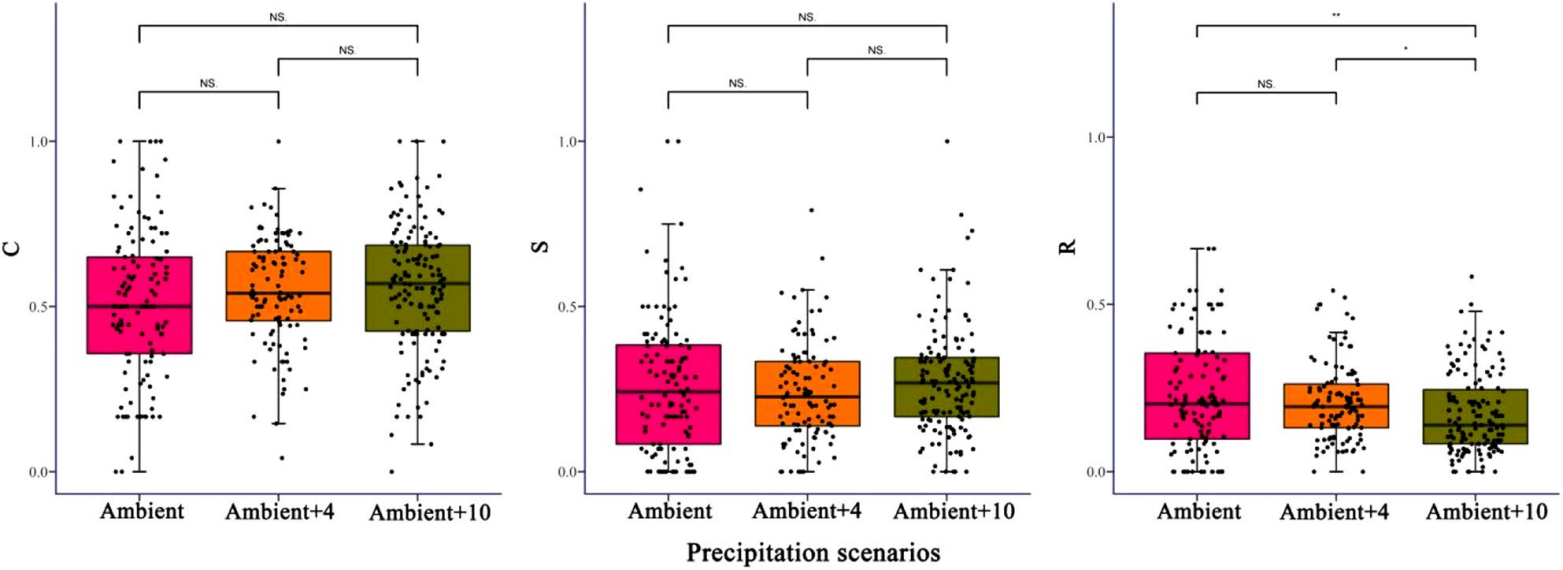
Changes in the position of plant communities along the axes of competitiveness (C), stress tolerance (S), and ruderality (R).

Plant communities shifted from the CSR to C/CSR classification in 2018 and 2019 (Fig. 4), with a clear community trend towards a competitor strategy, but the change in stress tolerance was weak. Moreover, precipitation increased the CSR distance that the CSR distance of Ambient between 2018 and 2019 was 0.18, while the Ambient + 4 and Ambient + 10 were 0.22 and 0.24. With the increase in precipitation in 2019, the Ambient was SC/CSR, but Ambient + 4 and Ambient + 10 were C/CSR (Fig. 4). Ruderals in the plant community significantly decreased, but competitors and stress-tolerant plants increased, although the change was not significant (Fig. 5).

## Discussion

### Effects of increased precipitation on the plant community

The increase in precipitation promoted the richness of sown species and resource-demanding plants, which was inconsistent with the general concept that climate change seriously threatens species diversity. Thus, increased precipitation in arid or semiarid regions such as Beijing could favour increased plant diversity, which is an opportunity for regions that experience more precipitation in the future. Jones et al.’s^46^ research in native tallgrass prairie also found that precipitation change increased species richness of forbs. They may have found this results as the study watered the plants in when there was no rain, increasing the total amount and frequency of precipitation. However, extreme precipitation resulting from climate change may have a greater impact on the diversity of plant communities^47^, and compound events such as floods and warmer temperatures will pose even more hazards^48,49^. Due to the limited conditions, our study did not simulate extreme floods, which can be simulated by controlling moisture conditions in specific plots or enclosed-top chambers in future research. On the other hand, precipitation only increased the species richness but had no significant effect on the dry matter content of the plant community, possibly because the net primary productivity was distributed more belowground than aboveground as precipitation increased^50^. However, precipitation change may strongly affect the water accumulation of plants, and fresh weight is important to reveal the impact of precipitation change on spontaneous plants and sown plants, which should be tested, and worth study in the near future.

Considering the long-term trend noted in Novakovskiy and Panyukov^39^, the change in the adaptive strategy of the plant community in the first two years was consistent with their noted change, and increased precipitation accelerated the process of succession such that the community accelerated away from the ruderal strategy. Ruderals in the first year occupied the area rapidly due to their capacity for rapid seedling establishment and growth^51^, but competitors dominated in the second year,thus, the plant community may tend towards stress tolerance in the future. The increase in precipitation significantly changed the adaptive strategy of the plant community and accelerated the rate of community succession, but the direction of succession was not benefical to the stability of plant community because the ecological strategy of natural grassland near Beijing was SC/CSR^52^. Precipitation promoted the growth of plant species, ruderals lost their advantage under minimal disturbance, and the community tended to compete more for limited water and other resources, which may inhibit long-term stability and sustainability of the community and means it require more human maintenance and environmental stress, such as pruning and drought, to ensure the sustainable landscape. Global change can either reshuffle species hierarchies or further favour already dominant species^53^. In addition, our research was conducted over two years, and long-term observations are essential to better understanding the dynamism caused by precipitation changes.

Our research considered spontaneous plants from the start, indicating that they have great potential to increase diversity, and we further divided them into resource-demanding plants and invasive plants. Many resource-demanding plants are stress tolerant and support persistent species-rich plant communities^40^. Efforts have been conducted in many countries to protect the resource-demanding spontaneous plants, and their value has been recognized by an increasing number of people. Consensus has grown related to removing only invasive plants in urban green spaces^54–56^, as spontaneous plants are an important part of the plant community; in addition, our study demonstrated that increased precipitation could lead to an increase in invasive plants (mainly alien species) and restrain the growth of sown plants, which may challenge the maintenance of urban vegetation in the near future.

### Precipitation’s interaction with design models and substrates

Plant design models have significant interactions with precipitation; thus, we should consider the fitness of community structure under climate change. The three-layer model proposed by Hitchmough^25^ was applicable not only in the UK but also in China, which has high diversity, dry matter content and weed resistance, and the three-layer model based on native grasslands was almost the same as this pattern; however, the single-layer model had exactly the opposite effect. Our results supported the concept that herbaceous vegetation with a three-layer structure is more resilient to future precipitation increases. Dissimilarity in different layers may favour the coexistence of rare species with dominant species by decreasing interspecific competition^57^, which could support more species and coverage, and the bare ground in single and double layers leads to the colonization of invasive weeds.

Intensive management in the field results in fewer spontaneous plants in the topsoil and subsoil. In contrast, the diversity of sand in this study was low and contributed to many weeds, which was inconsistent with the results of previous studies showing that sand mulch reduced the emergence of sown plants as well as weeds^31^, and this result may have occurred because the sand in the experiment was not sterilized. In addition, wildflower turf was nearly weed-free in the first two years and had high species richness and aesthetic value in the first year but low richness in the second year because of competition.

There was no significant interaction between substrate and precipitation change, but when the substrate was combined with the plant design model and precipitation, it had an impact on the species richness, indicating that there was a compound effect, however, the mechanism of this effect was not clear. Therefore, in order to cope with precipitation changes and increasing the richness of sown plants and resource-demanding plants, we should give priority to the planting design of the community, then use the appropriate substrate. Moreover, the comprehensive effects of multiple factors should be considered in planting design for climate change.

## Conclusion

Future precipitation increases were simulated by irrigation, and interestingly, we found that there was a significant interaction between precipitation and substrates, especially in the design models. In practical projects, planting design model is easier to achieve than the substrates, and we should focus on community structure in precipitation-sensitive areas or weedy areas at the beginning of the design, and supplemented by substrate improvement. Increased precipitation promoted the richness of sown plants and resource-demanding plants, which indicated the opportunity for climate change. Under the background of climate change, we should differentiate the spontaneous plants and protect resource-demanding species, which is benefit to increasing community diversity and stability. And increase in precipitation also accelerated community succession such that the functional strategy of these plants was significantly different from that of ruderals and tend to competitor strategy, which is detrimental for community stability and sustainability, and environmental stress help to cope with this change. Besides, we should pay more attention to the succession process of communities. Moreover, of the different structure, the three-layer structure had the highest species richness and least invasive plants. Thus, a multiple layer structure is recommended for planting designs that account for precipitation changes, and the species ratio of plant communities could be inspired from native vegetation.

### Supplementary Information

Supplementary Information.

## Data Availability

The raw data and supplementary information that support the findings of this study are openly available in Baidu Netdisk at https://pan.baidu.com/s/1n5IXnPdUbTGfMj28DVQO9g?pwd=1234.
