# Supplementary material for: The effects of precipitation change on urban meadows in different design models and substrates
Source: Sci Rep. 2023 Nov 23;13:20592. doi: 10.1038/s41598-023-44974-y (PMC10667351; doi:10.1038/s41598-023-44974-y)
Supplement: Supplementary file 1 — Supplementary Information. [file 41598_2023_44974_MOESM1_ESM.docx]

**Supplementary information availability statement：**

The data and supplementary information that support the findings of this study are openly available in Baidu Netdisk at https://pan.baidu.com/s/1n5IXnPdUbTGfMj28DVQO9g?pwd=1234.
